# Reconfigurable In-Sensor Computing Memristor for Olfactory SNN and Reservoir Hybrid Neuromorphic Computing

**DOI:** 10.34133/research.1071

**Published:** 2026-02-03

**Authors:** Lin Lu, Jinhao Zhang, Qingxin Chen, Jialin Meng, Yongjin Zou, Yilin Wang, Tianyu Wang

**Affiliations:** ^1^ Shandong Key Laboratory of Next-Generation Semiconductor Technology and Systems, School of Integrated Circuits, Shandong University, Jinan 250100, China;Suzhou Research Institute, Shandong University, Suzhou 215123, China.; ^2^ National International Innovation Center, Shanghai 201203, China.; ^3^ Key Laboratory of Computational Neuroscience and Brain-Inspired Intelligence (Fudan University), Ministry of Education, Shanghai 200433, China.; ^4^ Guangxi Key Laboratory of Information Materials, Guilin University of Electronic Technology, Guilin 541004, China.; ^5^ State Key Laboratory of Crystal Materials, Shandong University, Jinan 250100, China.

## Abstract

Traditional gas sensing systems are facing efficiency challenges due to physically separated von Neumann architectures, making the construction of in-sensor computing neuromorphic olfactory systems urgently needed for low-power and low-latency scenarios. In this study, a reconfigurable neuromorphic heterostructure memristor based on MXene@SnS_2_@PANI and an in-sensor computing olfactory system were proposed. Notably, the reconfigurable neuromorphic olfactory electronics differ fundamentally from conventional sensors. Specifically, the memristor’s circuit architecture supports both synaptic and neuronal computational functions, enabling reconfigurable responses to both electrical and gas stimuli within a single device, which substantially minimizes circuit complexity. Through modulation of the energy band under both gas and electrical signals, the device achieves reconfigurable neuromorphic computing features supporting both volatile and nonvolatile conductance updates. Under electrical stimulation, it demonstrates integrate-and-fire neuronal dynamics for gas flow recognition via a spiking neural network. Under gas exposure, neuromorphic synaptic behaviors are realized, enabling gas concentration identification through reservoir computing. The system has been successfully implemented for real-time hazardous gas monitoring and automated ventilation control, paving the way for next-generation neuromorphic intelligent sensing systems.

## Introduction

With the rapid advancements in Internet of Things (IoT) and artificial intelligence (AI) technologies, biological sensory systems have emerged as high-efficiency computing architecture. The human sensory nervous system exhibits low-power information sensing and processing capability with remarkable efficiency and minimal latency, promoting the development of neuromorphic biomimetic sensory systems [1–5]. These sensory systems act as bridges, transforming external stimuli into bioelectrical signals that connect the external environment with the internal nervous system, enabling vertebrates to perceive and interact with complex and dynamic environments [6–9]. However, traditional artificial sensory electronic systems comprise physically separated components of sensors, analog-to-digital converters, memory, and processing units, resulting in time delays and excessive energy consumption. Different from conventional reported hardware components, biological in-sensor computing systems offer an efficient computational model by integrating sensing, memory, and processing functions within the same synaptic units [10–14]. Therefore, it is in urgent need to develop in-sensor computing neuromorphic hardware for low power consumption information perception and computation with simplified architecture.

By drawing inspiration from biological sensory principles, artificial synaptic electronic devices exhibit promising multisensory functions for neuromorphic computing platforms [15–21]. Although various biologically inspired tactile sensing and visual systems have been developed, neuromorphic olfactory systems remain in nascent stages [22–25]. Olfactory systems play important roles in detecting environmental information and assessing the intensity and even toxicity of odors. However, traditional gas sensors can only convert gas signals into electrical signals, which need to upload information to cloud for recognizing and analyzing detailed gas information with huge power consumption [26–29]. To analyze gas flow rate and concentration, a gas sensor system integrates different circuit elements and AI algorithms (reservoir computing [RC] network and spiking neural network [SNN]), which leads to difficulties in circuit design and high-density integration [30]. Reconfigurable electronics, one element with multiple functions of synaptic plasticity and neuron functions, provide a potential path to address this issue [31–33]. Therefore, designing a reconfigurable olfactory neuromorphic computing system is considered as a possible approach for next-generation neuron–synapse neuromorphic computing tasks.

In this study, a reconfigurable artificial olfactory system that integrates both receptor neuron and synaptic characteristics was proposed for intelligent gas detection. It is important to emphasize that the reconfigurable neuromorphic olfactory electronics differ fundamentally from traditional sensors, primarily through the incorporation of a memristor-based design enabling dynamic circuit reconfiguration. Specifically, the circuit topology of the memristor allows a single device to implement neuromorphic operations—functioning as both synapses and neurons—with switchable capacity between gas and electrical modes, thereby markedly reducing circuit complexity. This system could realize both RC and SNN computing under gas and electrical mode. Based on the proposed heterostructure structure of MXene@SnS_2_@PANI, inherent defects of the materials were effectively passivated and the distribution of carriers was finely regulated. Gas signals could initiate excitatory or inhibitory responses of the postsynaptic membrane to external chemical stimuli, allowing for precise capture and analysis of the spatiotemporal characteristics by the RC network. Through meticulous energy band design, we have achieved a reconfigurable memory effect for both gas signals and electrical signals at the synaptic and neuronal levels, resulting in both RC and SNN computing under gas and electrical mode. Furthermore, the algorithmic advantages of RC and SNN computing were incorporated into a single device circuit, thereby constructing an intelligent in-sensor computing neuromorphic system for artificial olfaction. Real-time detection, processing, and warning processing of NH_3_ information were implemented in neuromorphic hardware. This reconfigurable in-sensor computing system lays a solid foundation for the development of future neuromorphic intelligent sensing applications.

## Results and Discussion

**Fig. 1.**
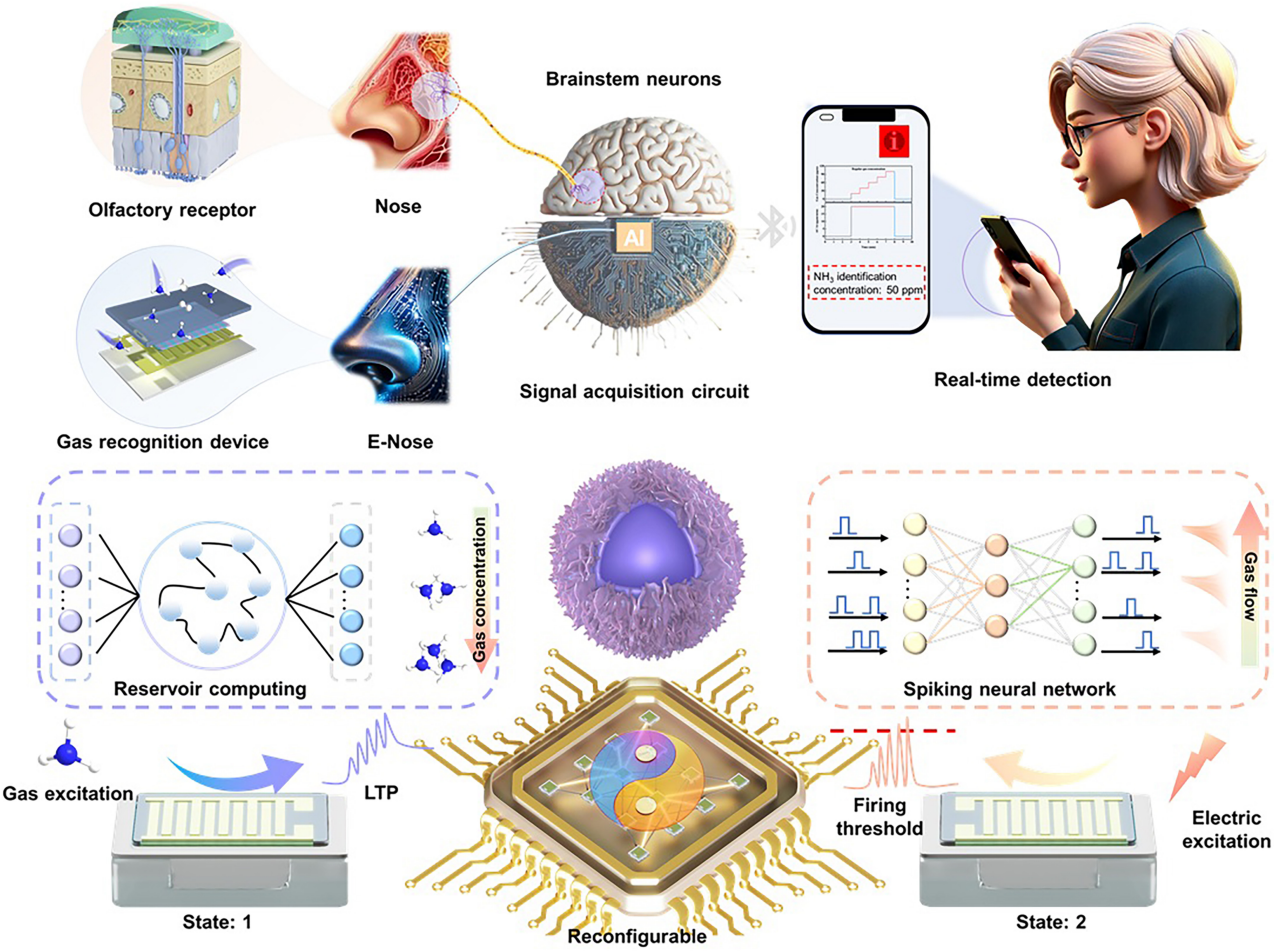
Schematic of reconfigurable neuromorphic intelligent olfactory system based on MXene@SnS_2_@PANI composites. Bio-inspired electronic nose construct artificial olfactory system, which shows potential in detecting, processing, analyzing and storage gas information. By designing energy band of synaptic olfactory device, reconfigurable characteristics of volatile and nonvolatile conductance response was achieved by changing gas and electrical stimulus. The electrical pulse could induce integrate-and-fire neural function for SNN computing and gas pulse could induce synaptic plasticity for reservoir computing (RC), paving the way for constructing intelligent warning system.

This device integrates principles of biological neuromorphic systems to facilitate the interaction of multisensory information. Its design philosophy draws upon the mechanisms of neural excitability regulation induced by human respiratory behaviors. Through an in-depth observation of human behavioral patterns (as illustrated in Fig. 1), we found that olfactory stimuli are initially captured by olfactory cells in the nasal cavity, which convert gas molecules into electrical signals. These signals subsequently induce neural excitations that are transmitted to the cerebral cortex, exhibiting a distinct excitatory characteristic. When biological olfactory receptors capture an odor, chemical reactions between neurons trigger the generation of electrical signals [34,35]. Subsequently, these signals are transmitted and processed via the glomeruli and olfactory bulbs, ultimately arriving at the olfactory cortex of the brain for recognition. In our meticulously designed intelligent olfactory system (Fig. 1), we employed an innovative MXene@SnS_2_@PANI sensor capable of simulating the functional attributes of an electronic nose, enabling precise detection of NH_3_ and variations in airflow [36,37]. MXene@SnS_2_@PANI composites were prepared as shown in Fig. S1. The sensor converts different concentrations of NH_3_ into corresponding electrical current signals, effectively simulating the process of neural excitation, which is then processed within the circuitry chip. To further enhance the intelligence of the system, we integrated both SNN and RC for training, successfully establishing a reconfigurable neuromorphic intelligent olfactory system. When this system detects elevated concentrations of NH_3_, it automatically triggers an alarm function and activates the exhaust fan to reduce the indoor NH_3_ levels. Additionally, the system is capable of real-time monitoring of NH_3_ concentration and gas flow rates, transmitting this information to a smartphone via Bluetooth technology, thereby achieving instantaneous monitoring and feedback of gas concentrations.

The crystalline structures of MXene@SnS_2_@PANI and MXene were characterized using x-ray diffraction (XRD), with the results presented in Fig. S3. MXene exhibited a characteristic and prominent diffraction peak at a 2*θ* value of 6.41°, corresponding to its (002) crystallographic plane [38]. The XRD spectrum of MXene@SnS_2_@PANI revealed distinctive diffraction peaks at 31.41° and 39.33°, which correspond to the (101) and (102) crystallographic planes of SnS_2_ (JCPDS card number 23-0677) [39], further confirming the successful synthesis of the MXene@SnS_2_@PANI composite material.

**Fig. 2.**
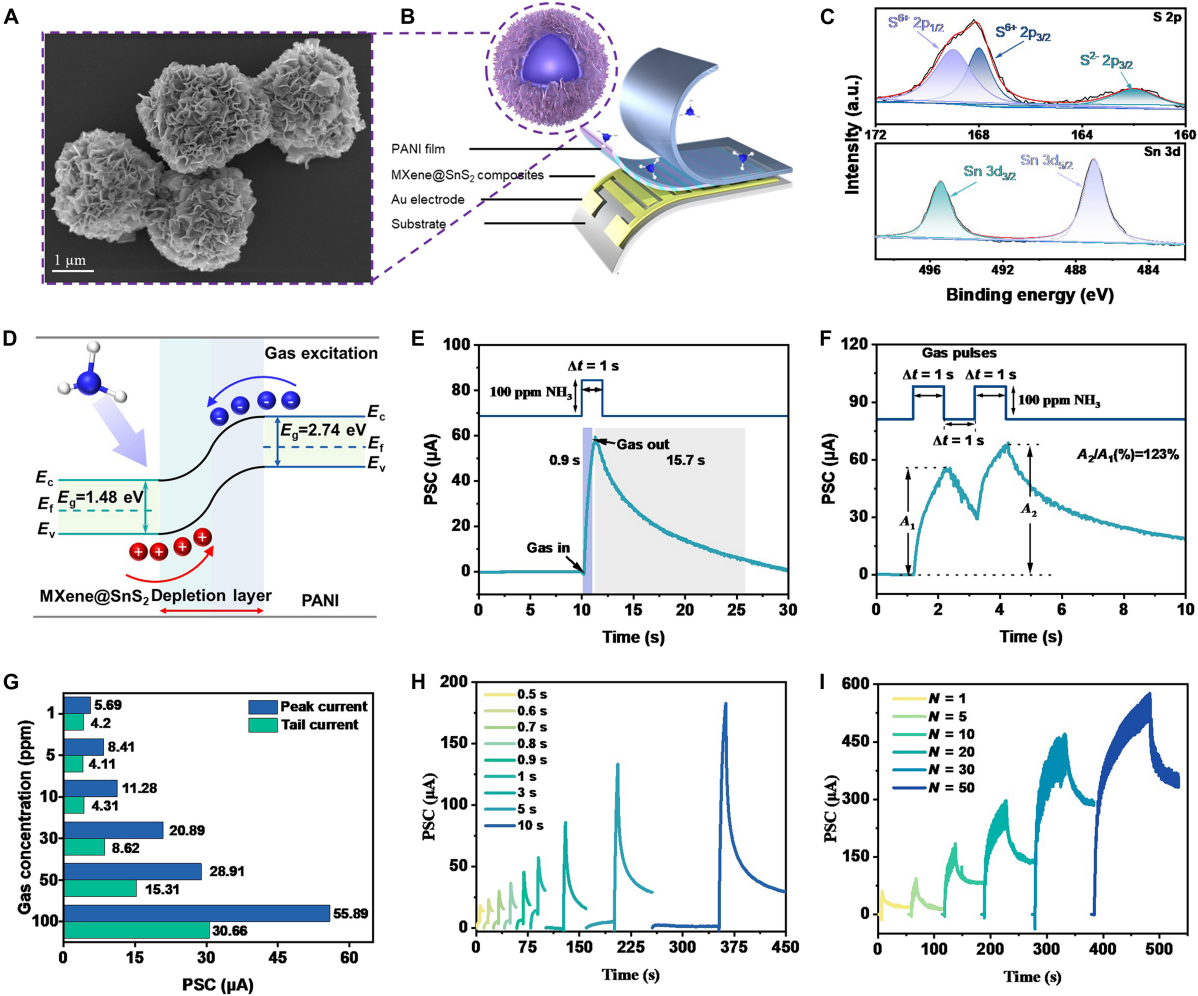
Gas-sensitive synaptic behavior of MXene@SnS_2_@PANI synapses. (A) SEM characterization map of MXene@SnS_2_@PANI composite. (B) Schematic diagram of gas-sensitive elements based on MXene@SnS_2_@PANI. (C) High-resolution XPS spectra of Sn and S elements. (D) Schematic diagram of energy bands in detection of NH_3_. (E) Recovery time test in response to 100 ppm NH_3_. (F) PPF curves of EPSC behavior triggered for 100 ppm NH_3_. (G) EPSC response and STP-to-LTP jump behavior triggered by different concentrations of NH_3_ (1, 5, 10, 30, 50, and 100 ppm). (H) Different pulse durations and (I) different number of pulses (NH_3_ concentration: 100 ppm, voltage = 8 V) triggering the EPSC response.

Figure 2A and Fig. S3A to D display scanning electron microscopy (SEM) images of MXene, PMMA@MXene, MXene@SnS_2_, and MXene@SnS_2_@PANI composites. Following the preparation process illustrated in Fig. S1, MXene nanosheets were systematically assembled into MXene@SnS_2_@PANI nanoflower balls with an approximate size of 1.8 μm. Subsequently, MXene@SnS_2_ and polyaniline (PANI) were sequentially spin-coated onto the surface of the gold interdigitated electrode to fabricate the gas-sensitive component depicted in Fig. 2B. MXene@SnS_2_ and PANI were successively spin-coated on the surface of the interpolation electrode (8 × 8 mm, finger width = 75 μm) to form films of 1 μm and 50 nm on the electrode surface, respectively (Fig. S2). The hollow structure of the material was further validated by the findings presented in Fig. S5A. Moreover, Fig. S5B and C demonstrate that the (101) and (102) crystallographic planes of SnS_2_ correlate with the XRD diffraction peaks, further substantiating the successful synthesis of the material. X-ray photoelectron spectroscopy (XPS) results (Fig. 2C and Fig. S7A to F) and energy dispersive spectroscopy (EDS) images (Fig. S6A to H) reveal the elemental composition of MXene@SnS_2_@PANI and confirm the formation of the heterojunction. Collectively, these results provide strong evidence for the successful fabrication of the NH_3_ gas-sensitive component based on MXene@SnS_2_@PANI. Figure S8 shows the atomic force microscopy (AFM) images of the spin-coated MXene@SnS_2_@PANI semiconductor layer. The results show that the prepared semiconductor layer has a large surface area and can effectively adsorb gases.

Subsequently, we conducted an in-depth investigation into the neuromorphic gas-sensing mechanisms and performance characteristics of the MXene@SnS_2_@PANI material, specifically focusing on its gas sensitivity and memory capabilities. As illustrated in Fig. 2D, we meticulously constructed a band structure diagram for the MXene@SnS_2_ and PANI materials. When the n-type MXene@SnS_2_ is combined with the p-type PANI, free electrons accumulate at the MXene@SnS_2_ interface, creating an electron potential barrier. Concurrently, holes accumulate at the interface of PANI, particularly near the Fermi level, thus establishing a hole potential barrier at the PANI end. The combined effects of these barriers lead to the formation of a thin depletion layer. PANI, a p-type semiconductor, possesses abundant amine and imine groups. Upon exposure to NH_3_ (an electron-donating gas), the molecules adsorb onto the PANI surface and donate electrons to its valence band. To neutralize this portion of electrons, n-type MXene@SnS_2_ begins to transfer holes to PANI, thereby forming a thick barrier that broadens the depletion layer. When the NH_3_ gas source is removed, the adsorbed NH_3_ molecules desorb from the surface of PANI. The donated electrons are then released back into the gas molecules during the desorption process.

In neuromorphic systems, synaptic and neuronal behaviors are distinguished by their fundamental computational roles. Synaptic plasticity refers to the activity-dependent modification of connection strength between neurons, which is the basis of learning and memory. This is typically characterized by phenomena such as short-term plasticity (STP), long-term plasticity (LTP), and paired-pulse facilitation (PPF). Neuronal dynamics, on the other hand, involve the integration of inputs and the generation of all-or-nothing output spikes (action potentials) when a threshold is exceeded, enabling information transmission and event-based coding. In this study, the characteristics of dynamic current synapses were deeply explored by analyzing the continuous injection of NH_3_ gas in the form of pulses. As clearly demonstrated in Fig. 2E, the prepared sensor exhibits excellent performance, with a response time and recovery time of only 0.9 and 15.7 s, respectively. Meanwhile, through the energy consumption calculation formula *E* = *V* × *I* × *t*, the power consumption of the gas sensor can be calculated to be 475.76 μJ. In Fig. 2F, we present the PSC (postsynaptic current) response of the device under the action of 2 consecutive NH_3_ pulses, where both the pulse interval (Δ*t*) and pulse width were set to 1 s and the NH_3_ concentration was kept at 100 parts per million (ppm). It is noteworthy that the PSC (*A*_2_) triggered by the second pulse was importantly increased compared to that triggered by the first pulse (*A*_1_), a phenomenon that vividly mimics the PPF behavior in biological synapses with PPF values (i.e., *A*_2_/*A*_1_) as high as 123%.

To further investigate the phenomenon, we measured the excitatory postsynaptic current (EPSC) responses induced by ammonia (NH_3_) concentrations of 1, 5, 10, 30, 50, and 100 ppm, and observed the transition from STP to LTP. The results indicate that both the peak current and the steady-state current exhibited an increasing trend with increasing NH_3_ concentration, suggesting that higher NH_3_ levels elicit stronger neural excitatory currents. Through the gradual augmentation of NH_3_ concentration, the transition from STP to LTP was distinctly observable. This transition not only reflects an enhancement in synaptic weight associated with LTP but also corresponds to a marked increase in the EPSC peak, thereby enhancing memory retention capabilities. Essentially, whether through elevated NH_3_ concentration, prolonged exposure duration, or enhanced light irradiation, an increase in the number of light-induced electron–hole pairs leads to a subsequent rise in EPSC levels. This transition from STP to LTP is visually represented in Fig. 2H and Fig. S9A to I. In our investigation of LTP dynamics, we also manipulated the pulse numbers of NH_3_ exposure to validate the LTP characteristics, as shown in Fig. 2I and Fig. S10A to F. This modulation holds important importance in simulating the learning and memory attributes of biological systems [40–43]. Our system demonstrated an important increase in both EPSC values and holding currents when subjected to multiple gas pulses. Specifically, following a series of 50 gas stimuli, our synapse exhibited substantial EPSC peaks of 576.57 μA, with a notable holding current of 344.49 μA sustained even 50 s after stimulation. These results effectively model the synaptic weight modulation process, achieving the transition from STP to LTP through iterative training, which closely resembles the behavior of native biological synapses. The transition between these 2 behavioral states can be attributed to the increased capture of electrons in contact with NH_3_, as the concentration of gas, pulse duration, or pulse frequency rises. Consequently, a longer time is required to neutralize the carriers within the depletion layer after the gas source is removed [44,45]. The unique selectivity of the prepared olfactory system for NH_3_ was determined by testing the STP/LTP characteristics of 100 ppm NO_2_, H_2_, CO_2_, and CO for different pulse numbers, and the results are shown in Figs. S11 to S14. The exceptional selectivity of the MXene@SnS_2_@PANI composite toward NH_3_ can be attributed to the synergistic effects of material composition and interfacial energy band alignment. PANI, as a p-type semiconductor, exhibits a strong affinity toward electron-donating gases such as NH_3_ due to its protonation–deprotonation mechanism. Upon exposure to NH_3_, the deprotonation of PANI leads to a decrease in hole carriers, thereby modulating the conductance. Meanwhile, the n-type SnS_2_ and highly conductive MXene framework facilitate efficient electron transfer, enhancing the sensitivity and response dynamics. The heterojunction formed at the MXene@SnS_2_/PANI interface further promotes charge separation and provides a tailored energy barrier that preferentially interacts with NH₃ molecules over other gases such as NO_2_, H_2_, CO_2_, and CO. This selective interaction is corroborated by the distinct STP/LTP behaviors observed under NH_3_ exposure compared to other gases (Figs. S11 to S14), underscoring the role of both chemical affinity and electronic structure in governing gas selectivity. For reducing gases (NH_3_ and H_2_), the primary interaction occurs with the p-type PANI component. As electron donors, these gases increase the electron concentration in PANI. This hole injection widens the depletion layer at the heterojunction interface, as described for NH₃. To maintain charge neutrality, this prompts an injection of holes from the n-type MXene@SnS_2_ into PANI. For the oxidizing gas (NO_2_), the interaction mechanism differs. NO_2_, a strong electron acceptor, primarily interacts with the n-type MXene@SnS_2_ component. Upon adsorption, NO_2_ molecules extract electrons from the conduction band of MXene@SnS_2_. To compensate for this electron loss, electrons are injected from the PANI side into MXene@SnS_2_. Crucially, the injection of electrons from PANI is equivalent to the injection of holes in the opposite direction (from MXene@SnS_2_ into PANI). This process similarly increases the positive space charge on the PANI side and the negative space charge on the MXene@SnS_2_ side, leading to a widening of the depletion layer. Therefore, although their chemical properties are opposite, both of these gases will trigger the same response.

**Fig. 3.**
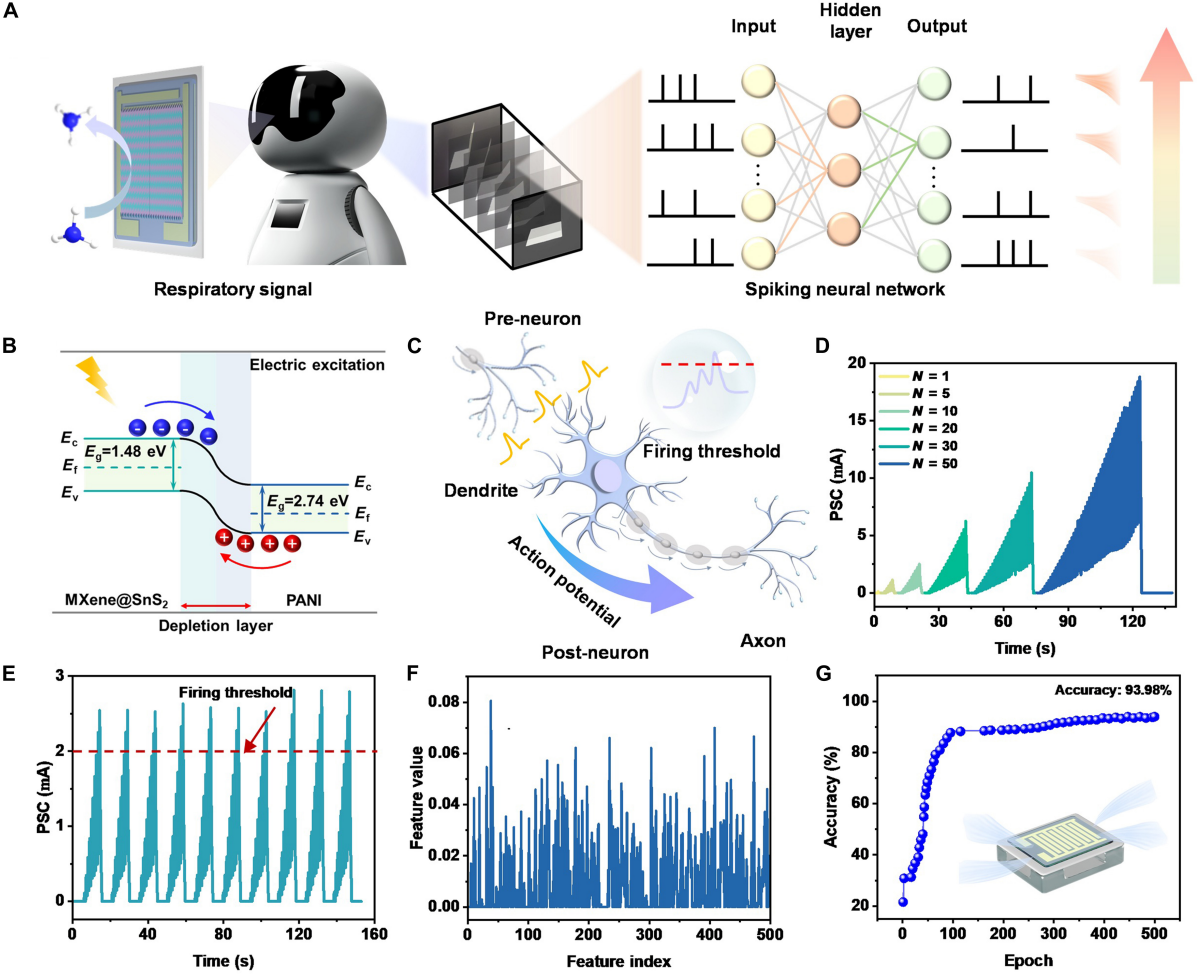
(A) Schematic diagram of SNN-resolved gas flow. (B) Schematic of electrical pulse energy band changes. (C) Schematic diagram of biological neurons with core integration-firing function. (D) Neural properties of different number of electrical pulses (*V* = −10 V). (E) Threshold properties of excitation neurons tested with 10 consecutive electric pulses. (F) Features of SNN. (G) SNN recognition accuracy.

To achieve precise modulation of neuronal weights, we have developed a novel approach that eliminates the need for gas stimulation, thereby establishing a solid foundation for neural plasticity. Figure 3A clearly illustrates the operational workflow of the constructed intelligent system during gas detection, which is influenced by both gas concentration and flow rate. Once a deviation is detected between the environmental flow rate and a nonleakage condition, the system, through training with an SNN, is capable of real-time monitoring and feedback of the gas flow information. In our system, the gas detection signal (concentration and flow rate) is first converted into a corresponding electrical current response by the MXene@SnS_2_@PANI sensor. This analog current signal is then preprocessed and encoded into a train of electrical pulses whose frequency and amplitude are proportional to the gas concentration and flow rate. These pulses serve as the input stimuli for the SNN training. Specifically, the SNN is trained to recognize temporal patterns of these pulse trains, which correspond to different gas flow scenarios (e.g., leak vs. no leak). This conversion from analog gas response to digital-like spike trains allows the system to leverage the event-driven and temporal coding capabilities of SNNs for efficient and low-power gas flow recognition.

Figure 3B presents a schematic diagram of the energy band under electrical pulsing. By utilizing electrical pulses as a substitute for NH_3_ stimulation, we observed that, in contrast to gas-induced energy bands, electrical stimulation facilitated the reverse migration of electrons and holes within the energy band. This migration phenomenon caused electrons from the n-type MXene@SnS_2_ surface to transfer to the PANI surface, while holes from the p-type PANI surface migrated toward the MXene@SnS_2_ surface. Consequently, this process prompted the depletion layer to transition from the broad state induced by NH_3_ stimulation back to its original narrow depletion layer state, further verifying the system’s reconfigurable characteristics.

Neurons, as the core units for information integration and transmission, are composed of a cell body, dendrites, and axons [46]. The generation of action potentials arises from the diffusion of ions across the cell membrane (as illustrated in Fig. 3C). When the current flowing through the neuron reaches a predetermined threshold, it transmits an integrated signal to the subsequent neuron. The reconfigurable neuro-olfactory system paves a new avenue for constructing artificial neurons based on a single device, leveraging the inherent characteristics of device pulse signal integration [47].

The basic memory behavior of the MXene@SnS_2_@PANI device was characterized under electrical stimulation. In the current–voltage (*I*–*V*) characteristic, a typical clamping hysteresis loop is a hallmark of a memristor. As shown in Fig. S15A and B, under continuous DC voltage sweep, the device presents a clear pinch hysteresis loop, which passes through the origin, confirming the memristor property of the device. The gradual change of conductance with continuous voltage scanning proves the analog programmability of the device, which is crucial for simulating synaptic weight updates. In addition, nonvolatile memory ability is a necessary condition for LTP, which is evaluated through retention and endurance tests. Figure S15C shows the retention characteristics of the device programmed to different conductance states (such as low resistance state and high resistance state) by applying appropriate voltage pulses. The state remains stable for more than 10^3^ s with minimal degradation, indicating excellent nonvolatile retention. These results provide fundamental electrical evidence for our device to operate as a reliable programmable memristor. The results of modulating the EPSC performance by adjusting voltage are presented in Fig. S16. When voltage varies within a range of 1 to 9 V (with a pulse width maintained at 0.3 s), an important enhancement in the current signal is observed. Furthermore, we optimized the pulse width of the electrical stimulation at a voltage of −10 V, as shown in Fig. S17. The state reconfigurability was further quantified by investigating the efficacy of electrical pulses in resetting the device conductance. The energy consumption during the electrical pulse was further calculated to be only 6.72 μJ through the energy consumption formula. As shown in Fig. 3D, a series of electrical pulses not only can excite the neuron but also effectively neutralize the persistent conductance change (LTP) induced by prior NH_3_ exposure. We systematically studied the recovery performance as a function of the applied voltage amplitude and pulse number. The results indicate that the completeness and speed of the state recovery can be finely controlled by the electrical stimulation parameters, providing a versatile handle to reconfigure the memristor’s state on demand. This electrically driven recovery process is the cornerstone that enables the cyclic operation of our system between gas sensing (writing) and electrical processing (resetting). This modulation technique is particularly important in simulating the learning and memory attributes of biological systems. When our system is stimulated by a series of continuous electrical pulses and reaches the preset threshold (*I* = 2 mA), the EPSC value increases, thereby transmitting the signal to the next neuron. The results of the simulated current tests are presented in Fig. 3E. In contrast to the graded, analog responses under gas stimulation, the device's behavior under electrical pulses exhibits defining features of neuronal operation. As shown in Fig. 3D and E, successive electrical pulses are integrated until a critical threshold is surpassed, triggering a sharp, fire-like response. This all-or-nothing, spiking behavior is the quintessential characteristic of neuronal firing. It enables the device to function as a spiking neuron within an SNN, performing tasks like gas flow recognition based on temporal integration and event-based coding, which is fundamentally different from the analog weight modulation used in synaptic operations. Figure 3F illustrates the processing of gas flow patterns into image-like features within the SNN framework. To classify the gas flow patterns, we constructed an SNN with a convolutional structure. By encoding more than 100 time bins using a threshold (rate), the waveform is encoded into a spike sequence, thereby obtaining the input dimension *N*_in_ = 100. SNN performs multiclass gas flow classification across *K* = 6 categories (no leakage plus 5 flow levels). This network is a 3-layer feedforward LIF model with 100-128-4 neurons. The LIF parameters are Δ*t* = 1 ms and *τ*_m_ = 10 ms. The synaptic weights were trained 100 times using proxy gradient backpropagation (learning rate, 1 × 10^−3^; batch size, 64). Each category contains 11 samples, which were collected using a flow controller at controlled flow rates ranging from 0.1 to 2.0 l/min. The gas chamber is cylindrical (with a volume of 50 cm^3^), and a fan is used to ensure uniform gas distribution. The response of the airflow currently detected by the device is transformed into a 2-dimensional time spectrum feature map by using the short-time Fourier transform for each input spike sequence (representing the gas flow rate over time). Then, these feature mappings are used as the input of the convolutional SNN for training and recognition. This method enables the network to simultaneously capture the time-domain and frequency-domain features of gas flow, thereby enhancing the recognition accuracy. Ten samples in the dataset are used for training, and the remaining one is used for testing. By employing a pulse neural network for training and gas flow recognition, the system achieves an accuracy rate of 93.98% (Fig. 3G) and a loss rate of 0.157% (Fig. S18), effectively demonstrating its exceptional performance.

**Fig. 4.**
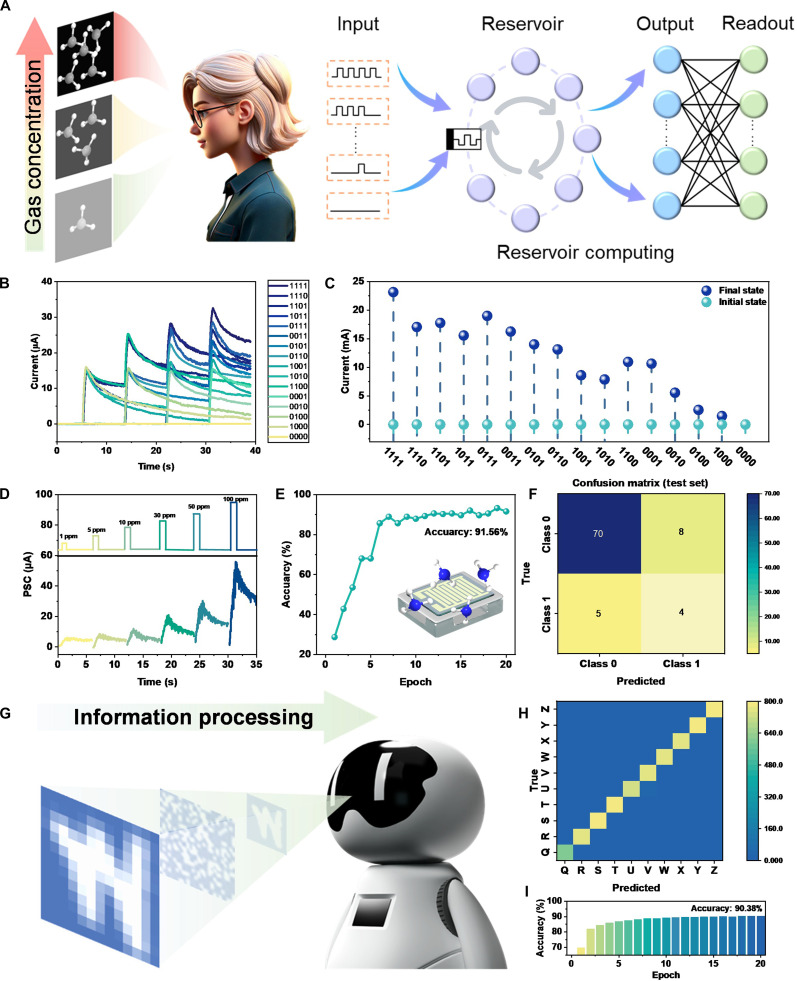
The intelligent olfactory detection based on RC is utilized to discriminate between different concentrations of ammonia (NH_3_). (A) A schematic representation of the recurrent neural network. (B) The encoding combinations based on binary states of “1” and “0”. (C) The steady-state current values. (D) Curves depicting gas concentrations ranging from 1 to 100 ppm. (E) Accuracy test of the RC. (F) The cross-validation plot of the RC. (G) Schematic diagram of RC optimization calculation. (H) “Q–Z” handwriting recognition verification. (I) Information processing recognition accuracy.

We have innovatively employed an RC-based olfactory system for the detection of ammonia (NH_3_) concentration. Figure 4A illustrates the schematic representation of our RC integration. In practical scenarios, gas concentration does not always increase monotonically; it may exhibit pulse-like fluctuations due to intermittent emission sources, ventilation cycles, or environmental disturbances. For instance, in industrial settings, gas leaks may occur in bursts, followed by dispersion or purging, leading to a recurring pulse train of concentration changes. Our RC-based system is designed to recognize such dynamic patterns, enabling robust detection and early warning even under nonmonotonic gas exposure conditions. This capability is crucial for real-world applications where gas presence is transient or cyclic, such as in smart manufacturing, hazardous material handling, or indoor air quality monitoring. To enhance the system’s flexibility and enable it to generate more diverse and adaptive responses during task execution, we employed gas pulses and nonpulses to represent the binary states of “1” and “0”, respectively. Sixteen different end-state currents are generated by using 16 different coding combinations as shown in Fig. 4B and C and Fig. S19. The current curves obtained under 5 consecutive pulse stimuli of different NH_3_ concentrations exhibit important differences in both peak and steady-state currents. The RC system was used to train for gas concentration recognition in response to well-defined gas pulses. Different concentrations of NH_3_ (1 to 100 ppm) diluted from the standard source were introduced into the test chamber in a series of pulses (Fig. 4D). Each pulse has a fixed duration and interval, enabling the device to exhibit its characteristic transient current response to each concentration. The resulting current–time data, which captures dynamic responses and steady-state values, is then used as a rich input signal for training the RC model. This method utilizes the temporal response of pulsed stimuli rather than just steady-state values, providing a high-dimensional dataset that enables the RC system to effectively learn and distinguish different gas concentrations with an accuracy of up to 91.56% (Fig. 4E). For the evaluation of gas detection performance using RC, the output classes were defined based on the presence or absence of gas flow. Class 0 was defined as the “no-flow” state (i.e., background environment without target gas flow), while Class 1 was defined as the “flow” state (i.e., presence of the target gas flow at a specific concentration). This binary classification task is essential for practical early-warning systems to distinguish between normal and hazardous conditions. The network’s performance was evaluated using 5-fold cross-validation, a robust technique to assess the generalization ability of the model by partitioning the dataset into 5 subsets (folds), iteratively using 4 for training and 1 for validation, and then averaging the results. This approach minimizes the bias and variance of the performance estimate. Following the training with NH_3_ concentrations ranging from 1 to 50 ppm, the results from folds 1 to 5 are presented in Fig. S20B to F. Experimental validation was subsequently conducted using 100 ppm NH_3_. The cross-validation results, shown in Fig. 4F, demonstrate the network’s capability to distinguish NH_3_ concentrations across all polarization states. The confusion matrices for the fold cross-validation (Fig. 4F and Fig. S20) show that the model achieved a high overall accuracy. It is noted that the classification accuracy for Class 0 (“no-flow”) is consistently higher than that for Class 1 (“flow”). This performance difference can be attributed to the greater variability in the gas adsorption–desorption dynamics and transient signal patterns under the “flow” condition compared to the more stable baseline of the “no-flow” state. Meanwhile, using this approach to simulate the activity of the biological nervous system, the RC is applied to sum 4 handwritten letter “W” pixel dots into one (Fig. 4G), which enhances the computational power and adaptive capability of the system. Figure 4H and Fig. S21 show the cross-validation diagram of “A to Z” 26-letter handwriting recognition, which enhances the computational ability and speed of the intelligent system by processing the gas information into different letters. We extended its application to handwriting recognition tasks, further demonstrating the powerful capability of our RC system in handling complex spatiotemporal information. This experiment aims to verify the multifunctionality of the system rather than optimize the recognition performance of different gas concentrations. Handwriting images (e.g., the pixel map of the letter “W” or the “A to Z” alphabet) were not directly input as gas pulses. Instead, the binary pixel values (0 for background, 1 for letter) of a flattened image sequence were used to control the timing of gas pulses. A gas pulse (using 100 ppm NH₃) was applied to the device to represent a pixel value of “1”, while no pulse (“0”) was applied for a pixel value of “0”. The resulting transient current response of the device to this predefined gas pulse sequence was recorded. This current response, which encodes the spatiotemporal dynamics of the device, served as the input signal to the reservoir computer for training and classification. In this paradigm, the reservoir computer was not recognizing the gas concentration itself, but rather the temporal signature of the device’s response to a fixed-concentration gas pulse sequence that encodes visual information. The system successfully achieved a recognition accuracy of 90.38% for the 26 letters (Fig. 4I), effectively processing gas-mapped information into different categories. This experiment serves as a compelling demonstration of the system's ability to perform complex classification tasks based on temporal patterns generated by gas pulses. The pulse-based encoding and RC training enable the system to recognize not only steady-state concentrations but also temporal patterns of gas exposure, mimicking real-world scenarios where gas signals may fluctuate intermittently.

**Fig. 5.**
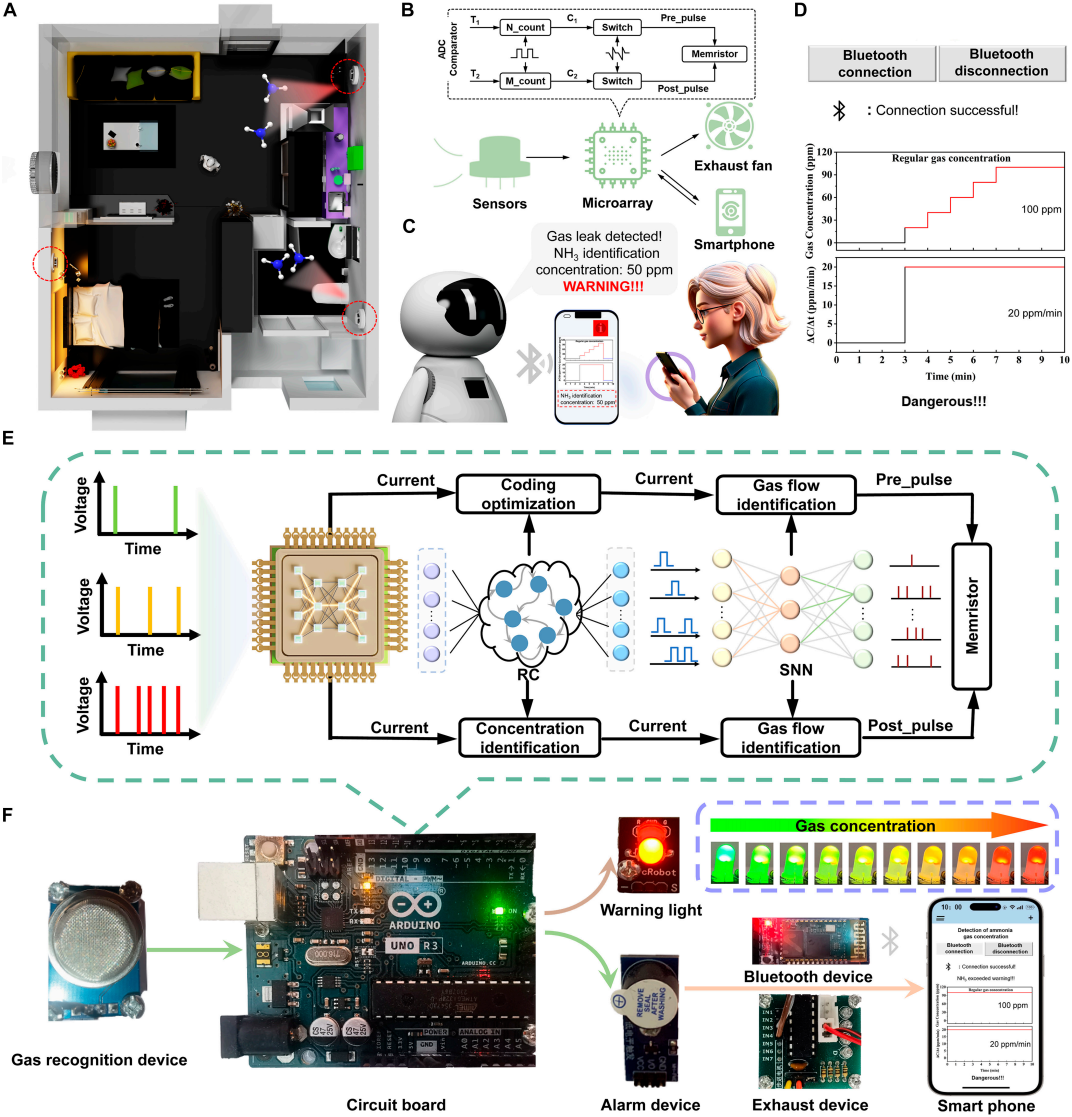
(A) Simulation of application scenarios for the reconfigurable neural network intelligent system. (B) Schematic diagram of the bio-inspired hardware system architecture based on MXene@SnS_2_@PANI neuromorphic devices, which includes a microarray, a sensing module, an exhaust fan for ventilation, a Bluetooth module for data transmission, and a signal transmission and conversion module between the neuromorphic matrix and the microarray core. (C) Schematic representation of the intelligent olfactory system transmitting data to a mobile phone via Bluetooth. (D) Display interface on the mobile phone. (E) Schematic diagram of the system combining SNN and RC processing gas signal reconfigurability. (F) Photograph of the hardware system.

Due to the compact nature of our intelligent olfactory system, we established an internal sensor network composed of multiple electronic noses and a mobile receiver, along with the development of a software application for data processing. Figure 5A illustrates the deployment of the intelligent olfactory system within a smart home environment as a safety monitoring system. Owing to its long operational lifespan and wireless communication capabilities, the sensor nodes can be distributed at any preferred location within the Bluetooth broadcast range, which can extend up to 100 m.

Figure 5B presents a schematic diagram of the biomimetic hardware system architecture based on MXene@SnS_2_@PANI neuromorphic devices, which includes a microarray, a sensing module, an exhaust fan, a Bluetooth module for data transmission, and signal transmission and conversion modules between the neuromorphic matrix and the microarray core. The collected data are processed by the chip and undergo neuromorphic processing before being transmitted via Bluetooth to the residents’ mobile phones, enabling real-time monitoring of the home environment from anywhere (Fig. 5C), with the mobile display shown in Fig. 5D and Fig. S22. Figure 5E and Fig. S23 present a detailed schematic diagram of the signal processing workflow within the hardware system after the target gas is detected. We integrated a single memristor into the circuit and conducted the test using a 5-V DC power supply as the input voltage. When this device detects pulsed gas, it works like a neural synapse. The sensor transmits the detected gas signal to the chip in the form of current, and then, through the use of RC, process optimization and gas concentration identification are carried out in sequence. The temporal dynamics of the response were analyzed by combining 2 RC networks to accurately identify the gas concentration. Subsequently, the same analog signal is encoded into a series of electrical pulses. These pulses are still sent into the SNN processing path in the form of current pulses. SNN integrates these pulses. If the integrated value exceeds the preset threshold, it indicates that a major event has occurred (for example, gas leakage), triggering an output spike. The final decision, such as activating the alarm or the exhaust fan, is made based on the coordinated output of the RC and SNN pathways. This dual-path architecture ensures powerful and intelligent gas detection and response. The actual physical representation of the system is depicted in Fig. 5F, where the addition of warning lights allows for color changes in response to varying NH_3_ concentrations.

## Conclusion

In summary, we have successfully demonstrated a reconfigurable artificial olfactory system based on various neuromorphic computing principles. It should be emphasized that reconfigurable neuromorphic olfactory electronics fundamentally differ from traditional sensors, as they achieve intelligent perception–computation integration via dynamic memristor networks. The MXene@SnS_2_@PANI reconfigurable neuromorphic memristive devices, fabricated through energy band design, are utilized to facilitate gas molecule capture and enable electronic interaction escape, thereby promoting typical synaptic behavior. Notably, the memristor-based circuit architecture allows the same device to perform both synaptic and neuronal computation, switching between gas and electrical modes, which greatly reduces circuit complexity. Furthermore, the synergistic effects of NH_3_ stimulation and electrical stimulation have allowed for the realization of synaptic reconfigurable characteristics, importantly reducing system complexity while enhancing recognition and computational efficiency. The system integrates multiple neuromorphic computing paradigms, including SNN and RC, within a single device to recognize gas flow and monitor real-time NH_3_ concentrations. This integration embodies the integrating and firing characteristics of neurons, thereby substantially decreasing circuit complexity. The reconfigurable neural network ensures more accurate detection outcomes, which holds important implications for early warning systems. We have constructed a reconfigurable neuromorphic hardware warning system based on these reconfigurable devices, capable of real-time monitoring of hazardous gas concentrations and enabling remote automatic control for alarms and ventilation. This advancement makes the development of future neuromorphic intelligent sensing systems feasible.

## Materials and Methods

### Preparation of hollow MXene microspheres

To prepare the hollow MXene microspheres, 3.2 g of lithium fluoride was uniformly dispersed in hydrochloric acid solution and stirred under heating in a water bath. Subsequently, 2 g of Ti_3_AlC_2_ was added to the reaction system, and the mixture was continuously stirred at 40 °C for 24 h. Upon completion of the reaction, the resulting product was dried at 60 °C overnight to obtain powdery MXene materials. A PMMA [poly(methyl methacrylate)] template sphere was then combined with the MXene in a mass ratio of 10:1, and the mixture was added to ultrapure water, followed by continuous stirring for 30 min. Through electrostatic adsorption, MXene successfully adhered to the surface of the PMMA spheres, which were then collected by centrifugation and dried to yield PMMA@MXene composite spheres.

### Preparation of MXene@SnS_2_ nanoflower ball films

The synthesis of MXene@SnS_2_ nanoflower balls was accomplished using a hydrothermal method. First, 100 mg of MXene spheres was placed in a mixed solvent consisting of 30 ml of ethanol and 10 ml of ethylene glycol, and the mixture was ultrasonicated for 30 min to facilitate thorough dispersion. Subsequently, 114 mg of thiourea and 105 mg of tin chloride pentahydrate were sequentially introduced into the dispersion and stirred at room temperature for 30 min to ensure uniform adherence of the tin source to the PMMA@MXene surface. The resulting mixture was then transferred to a high-pressure reactor and maintained at 150 °C for 24 h. After the reaction, the product was allowed to cool naturally to room temperature and was subjected to multiple washes with ethanol to remove impurities. Finally, the washed product underwent vacuum drying at 80 °C. The resulting powder sample was then subjected to annealing at 350 °C in an argon atmosphere for 1 h to facilitate the complete decomposition of PMMA, yielding MXene@SnS_2_ nanoflower balls. A small amount of the sample was dissolved in ethanol and applied via spin coating on the surface of a 10 mm × 10 mm interdigitated electrode to form a uniform functional film.

### Preparation of MXene@SnS_2_@PANI composite film sensors

The MXene@SnS_2_@PANI composite sensor device was prepared using a spin-coating method. Initially, 30 mg of PANI was dissolved in 5 ml of N,N-dimethylformamide. The prepared PANI solution was then drop-cast onto the surface of the MXene@SnS_2_ device and spin-coated at a speed of 5,000 rpm for 30 s. The device was subsequently placed in a ventilated area to dry naturally, resulting in the formation of the composite film sensor device.

### Device characterizations

The structure and composition of the device were determined by XRD, SEM, transmission electron microscopy, EDS, and XPS. All gas tests are measured at room temperature using a gas sensing test system (JF02F). All experiments were conducted at 25 ± 0.2 °C and approximately 45% relative humidity. A computer-controlled dynamic gas mixing device is adopted to enable various test gases to enter the gas chamber. Dilute the purchased dry standard gas to 500 ppm to obtain different concentrations of NH_3_, H_2_, CO_2_, CO, and NO_2_ required for the test.

## Data Availability

Data will be made available on request.
