# Supplementary material for: Reconfigurable In-Sensor Computing Memristor for Olfactory SNN and Reservoir Hybrid Neuromorphic Computing
Source: Research (Wash D C). 2026 Feb 3;9:1071. doi: 10.34133/research.1071 (PMC12864652; doi:10.34133/research.1071)
Supplement: Supplementary 1 — Materials and methods Figs. S1 to S23 [file research.1071.f1.docx]

**Supporting Information**

**Reconfigurable In-sensor Computing Memristor for Olfactory SNN and Reservoir Hybrid Neuromorphic Computing**

*Lin Lu^1^, Jinhao Zhang^1^, Qingxin Chen^1^, Jialin Meng^1,3,4*^, Yongjin Zou^2*^, Yilin Wang^1^, and Tianyu Wang^1,3,5*^*

*^1^Shandong Key Laboratory of Next-Generation Semiconductor Technology and Systems, School of Integrated Circuits, Shandong University, Jinan 250100, China; Suzhou Research Institute of Shandong University, Suzhou, 215123, China*

*^2^Guangxi Key Laboratory of Information Materials, Guilin University of Electronic Technology, Guilin 541004, China;*

*^3^**National International Innovation Center, Shanghai 201203, China;*

*^4^Key Laboratory of Computational Neuroscience and Brain-Inspired Intelligence (Fudan University), Ministry of Education, Shanghai 200433, P. R. China*

*^5^State Key Laboratory of Crystal Materials, Shandong University, Jinan, 250100, China*

**Email: jlmeng@sdu.edu.cn; zouy@guet.edu.cn;* [*tywang@sdu.edu.cn*](mailto:tywang@sdu.edu.cn)

**The supporting information file includes:**

**Figure S1.** Schematic of the preparation of MXene@SnS_2_@PANI composites.

**Figure S2.** Schematic diagram of film thickness and details of the intercalation electrode.

**Figure S3.** XRD patterns of MXene and MXene@SnS_2_@PANI composites

**Figure S4.** SEM images of (a) MXene, (b) PMMA@MXene, (c) MXene@SnS_2_ and (d) magnified image of MXene@SnS_2_.

**Figure S5.** (a) TEM morphology; (b) lattice streak pattern and (c) crystal diffraction rings of MXene@SnS_2_@PANI.

**Figure S6.** (a) HAADF image of MXene@SnS_2_@PANI. (b) Elemental map of MXene@SnS_2_@PANI showing selected regions including (c) O, (d) C, (e) S, (f) Ti, (g) N and (h) Sn.

**Figure S7.** XPS spectra of (a) MXene@SnS_2_@PANI and typical high-resolution XPS images of (b) N 1s, (c) O 1s, (d) C 1s, and (e) Ti 2p.

**Figure S8.** AFM mapping of the prepared gas-sensitive element.

**Figure S9.** Synaptic properties for different pulse widths (a) 0.5 s; (b) 0.6 s; (c) 0.7 s; (d) 0.8 s; (e) 0.9 s; (f) 1 s; (g) 3 s; (h) 5 s; (i) 10 s.

**Figure S10.** Characterization of LTP with different number of pulses. The number of pulses are (a) N=1; (b) N=5; (c) N=10; (d) N=20; (e) N=30; (f) N=50.

**Figure S11.** Different number of pulses (NO_2_ concentration: 100 ppm, VDS = 8 V) triggering the EPSC response.

**Figure S12.** Different number of pulses (H_2_ concentration: 100 ppm, VDS = 8 V) triggering the EPSC response.

**Figure S13.** Different number of pulses (CO_2_ concentration: 100 ppm, VDS = 8 V) triggering the EPSC response.

**Figure S14.** Different number of pulses (CO concentration: 100 ppm, VDS = 8 V) triggering the EPSC response.

**Figure S15.** (a) I-V curve test in linear coordinates, (b) I-V curve in logarithmic coordinates, (c) maintenance characteristics of high and low configurations.

**Figure S16.** Different voltage with a pulse width of 0.3 s.

**Figure S17.** Different pulse width test at －10V.

**Figure S18.** SNN loss rate curve.

**Figure S19.** The numbers "1" and "0" represent the five-degree repeatability statistical graph.

**Figure S20.** RC cross-validation training process.

**Figure S21.** Handwriting recognition of the letters "A-P".

**Figure** **S22.** Bluetooth detection display diagram.

**Figure S23.** Bluetooth detection display diagram.


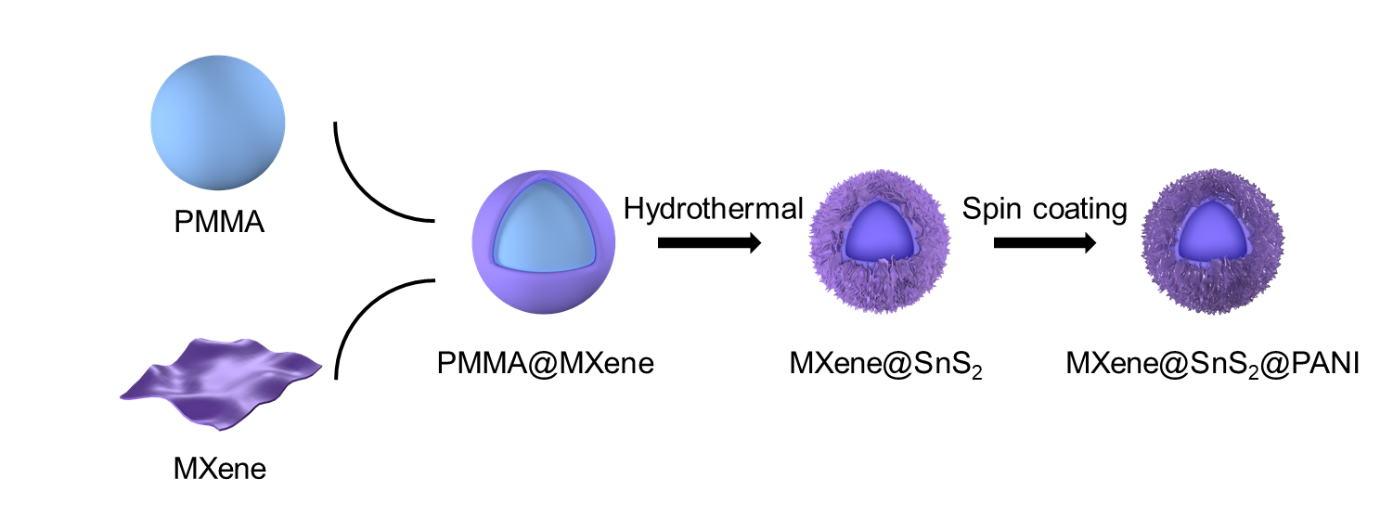


**Figure S1.** Schematic of the preparation of MXene@SnS_2_@PANI composites.


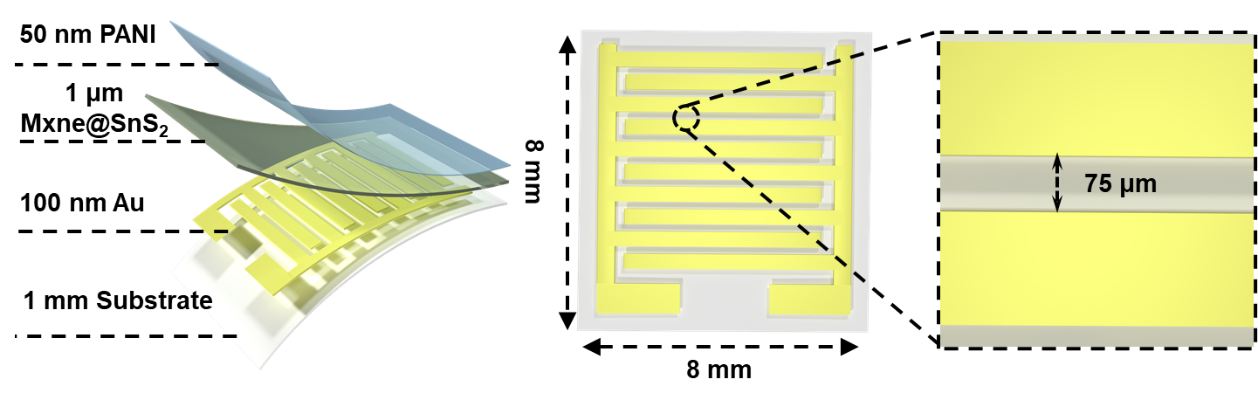


**Figure S2.** Schematic diagram of film thickness and details of the intercalation electrode.


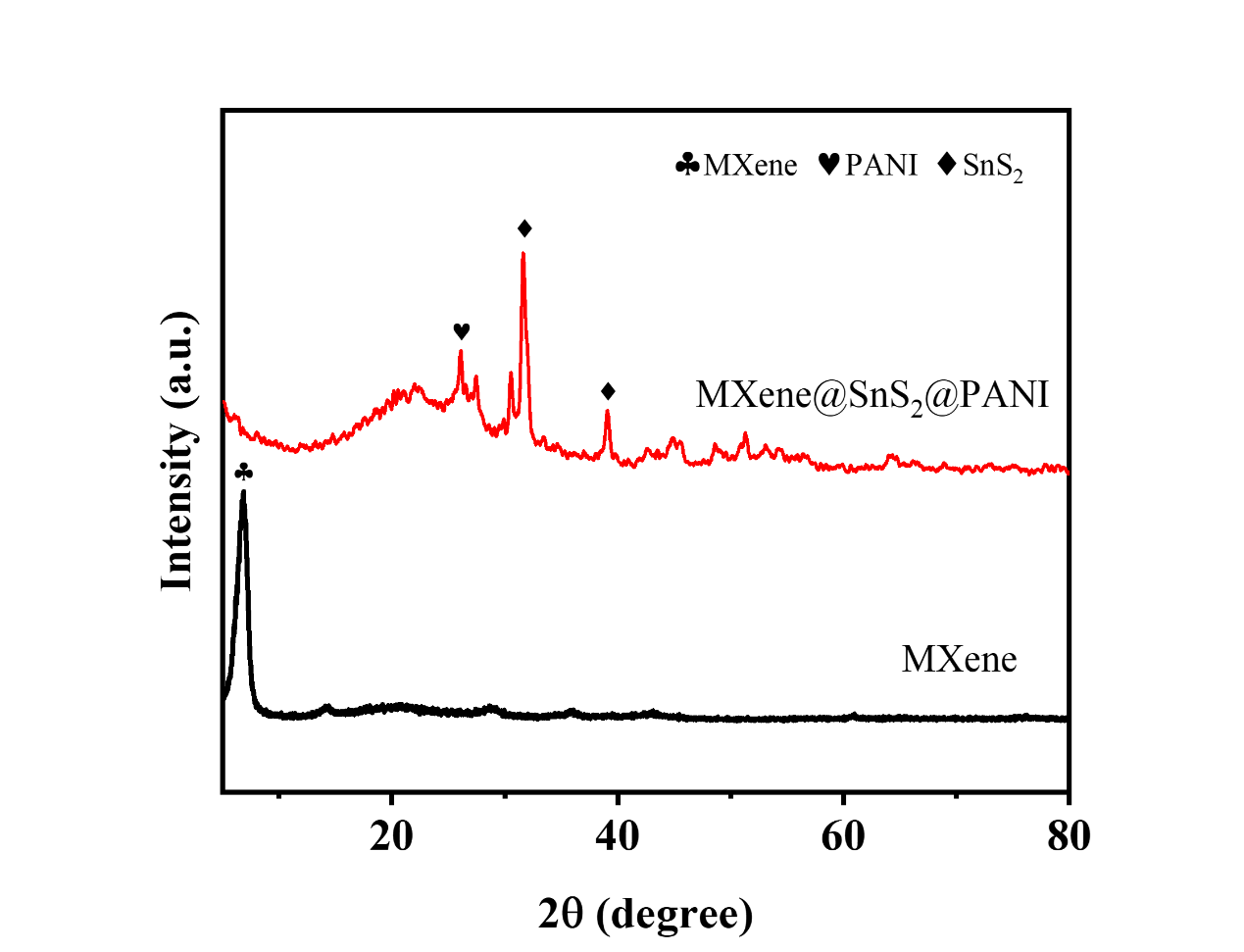


**Figure S3.** XRD patterns of MXene and MXene@SnS_2_@PANI composites.


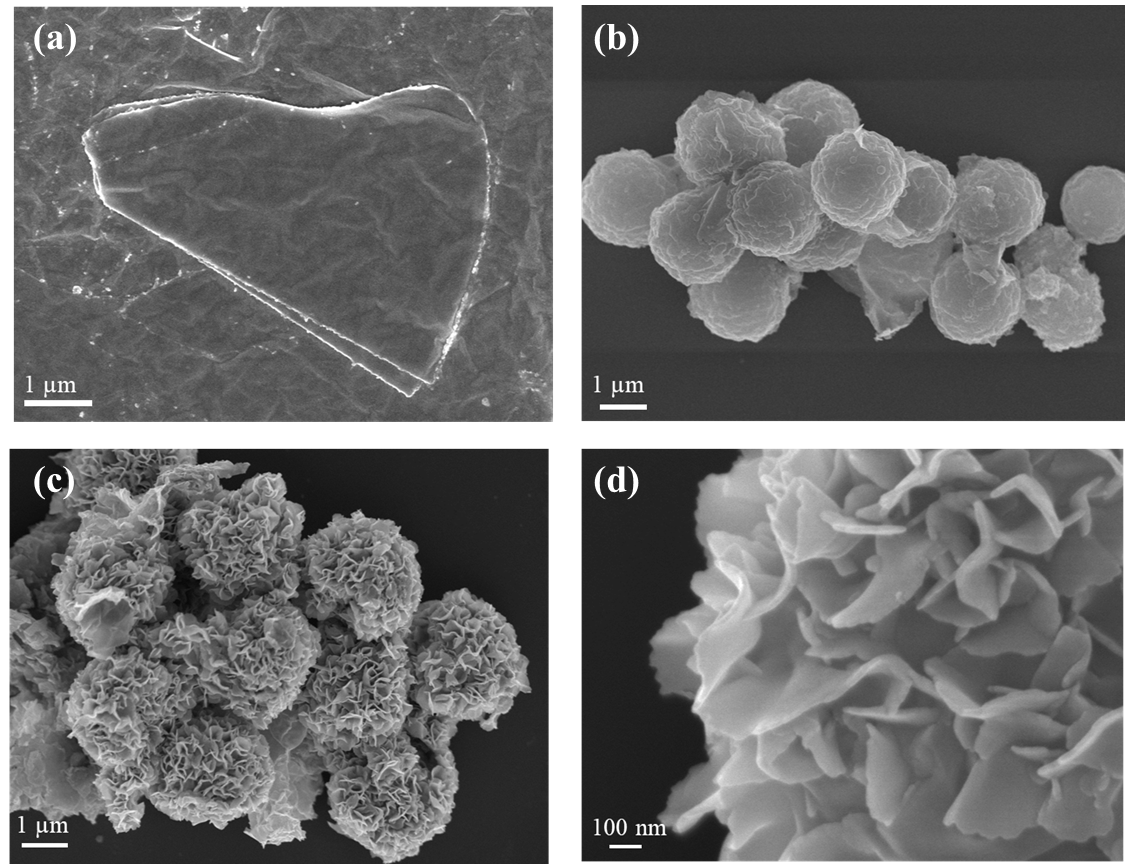


**Figure S4.** SEM images of (a) MXene, (b) PMMA@MXene, (c) MXene@SnS_2_ and (d) magnified image of MXene@SnS_2_.


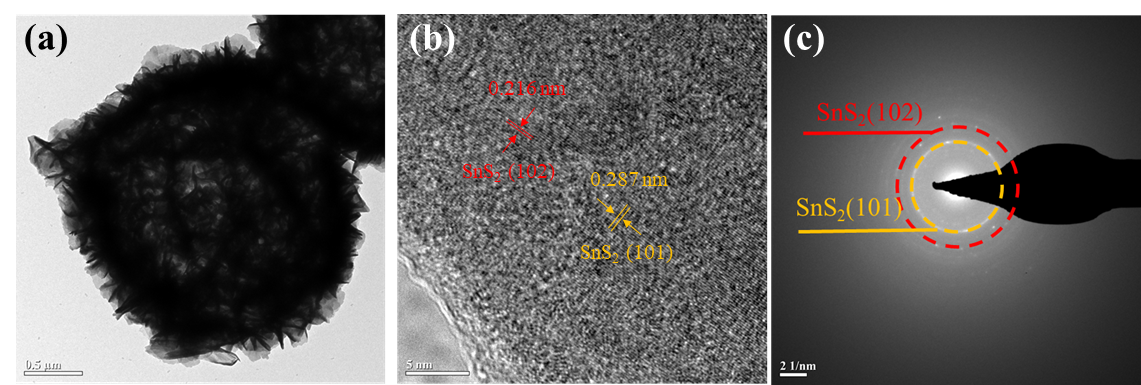


**Figure S5.** (a) TEM morphology; (b) lattice streak pattern and (c) crystal diffraction rings of MXene@SnS_2_@PANI.


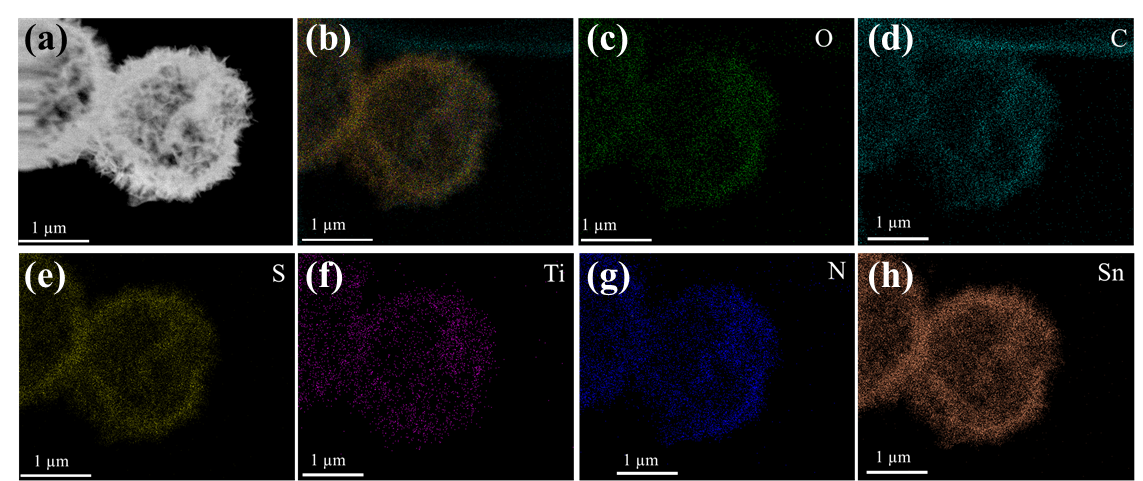


**Figure S6.** (a) HAADF image of MXene@SnS_2_@PANI. (b) Elemental map of MXene@SnS_2_@PANI showing selected regions including (c) O, (d) C, (e) S, (f) Ti, (g) N and (h) Sn.


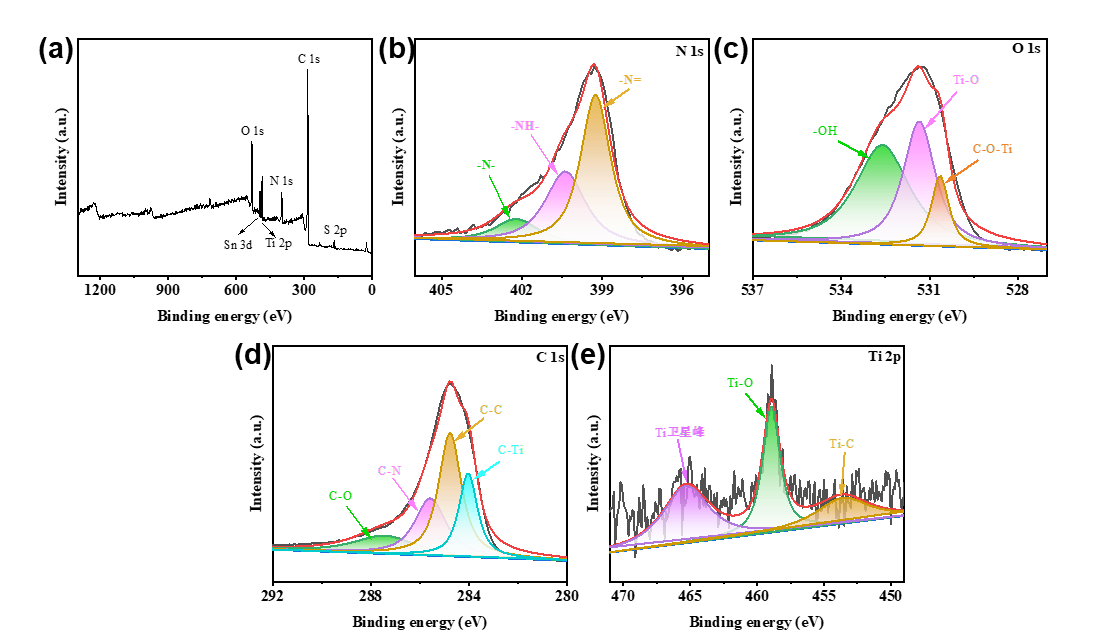


**Figure S7.** XPS spectra of (a) MXene@SnS_2_@PANI and typical high-resolution XPS images of (b) N 1s, (c) O 1s, (d) C 1s, and (e) Ti 2p.


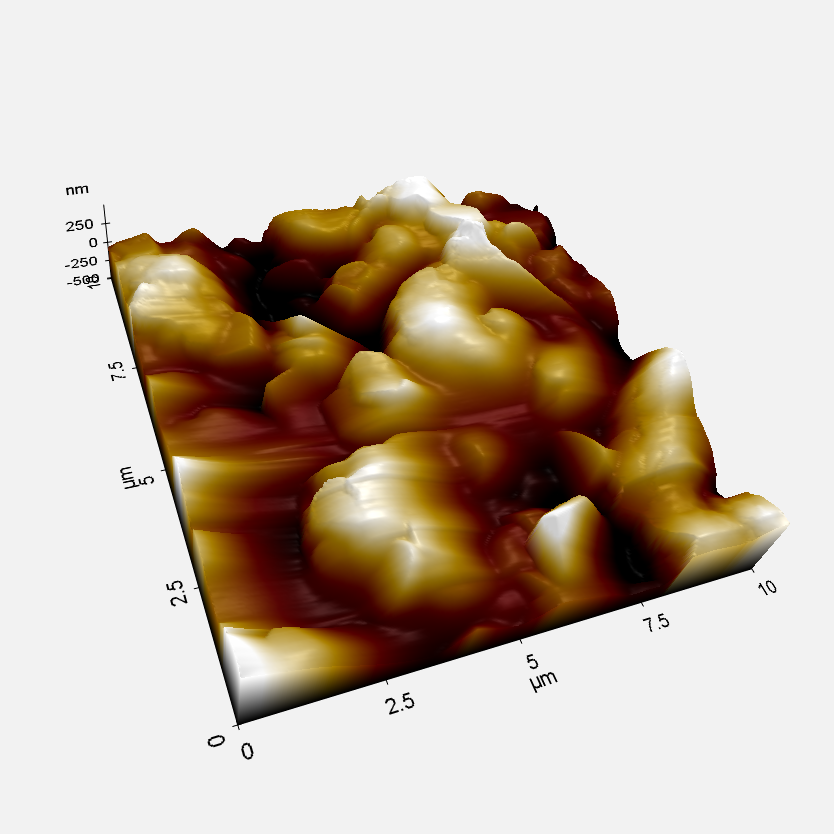


**Figure S8.** AFM mapping of the prepared gas-sensitive element.


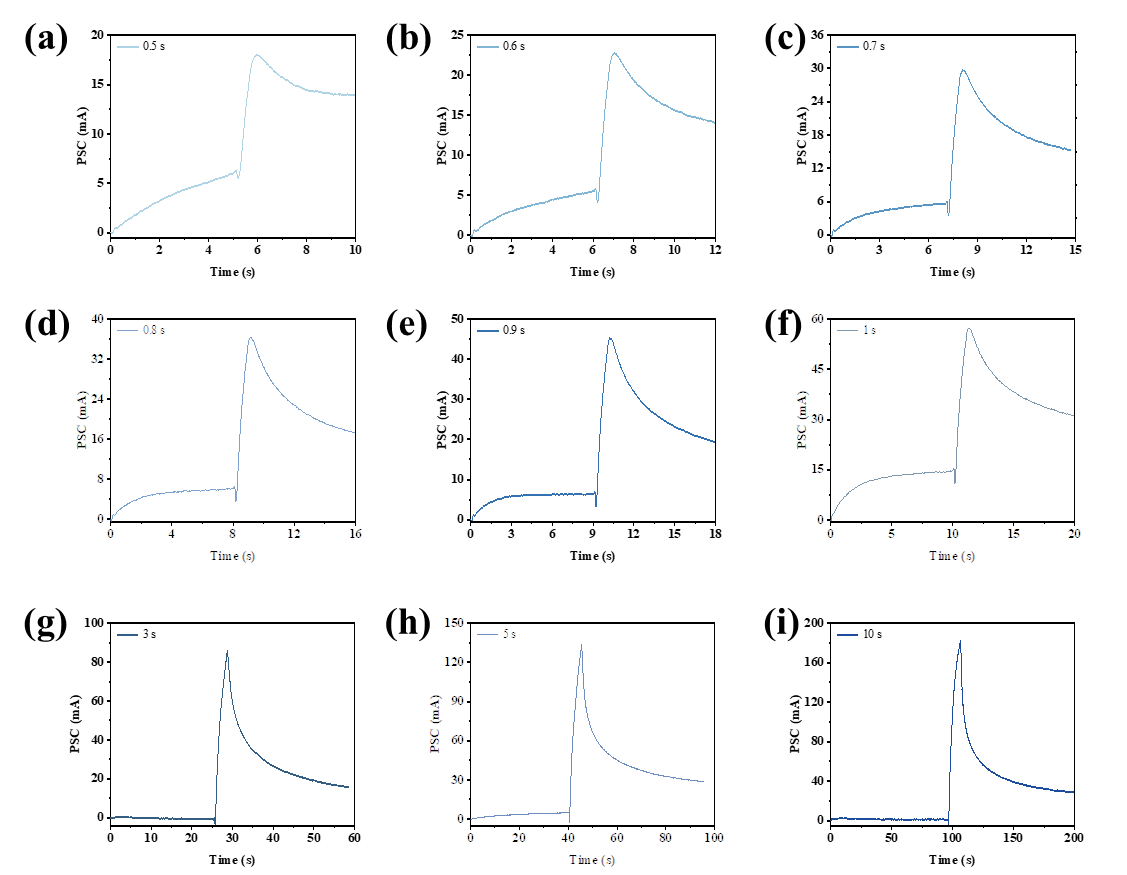


**Figure S9.** Synaptic properties for different pulse widths (a) 0.5s; (b) 0.6s; (c) 0.7s; (d) 0.8s; (e) 0.8s; (f) 1s; (g) 3s; (h) 5s; (i) 10s.


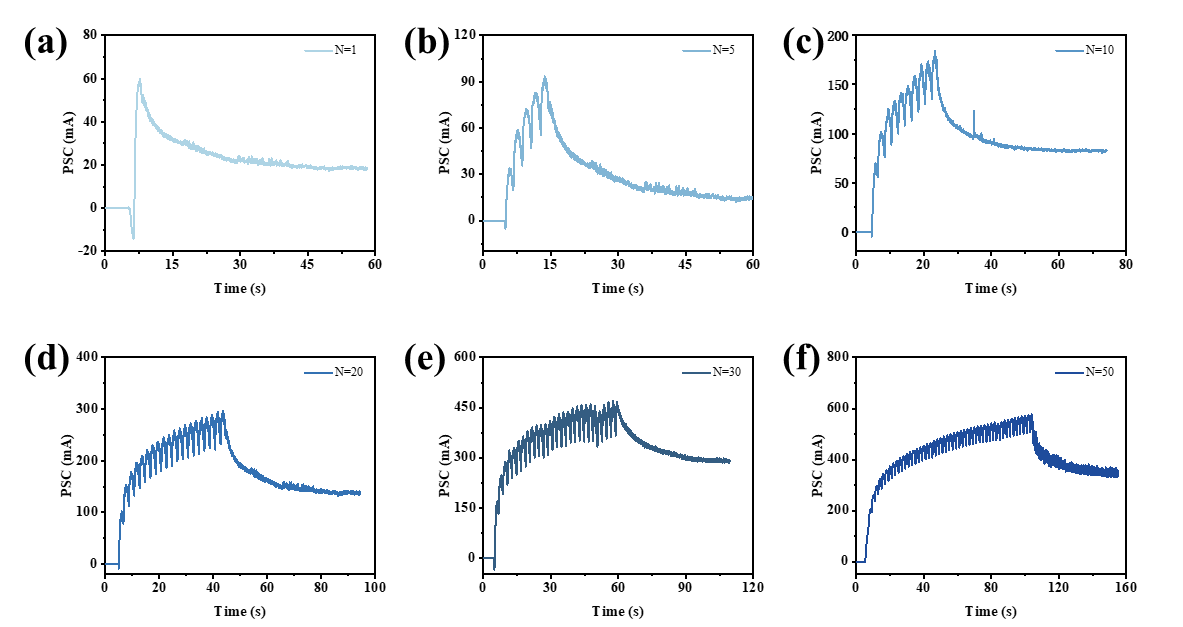


**Figure S10.** Characterisation of LTP with different number of pulses.The number of pulses are (a) N=1; (b) N=5; (c) N=10; (d) N=20; (e) N=30; (f) N=50.


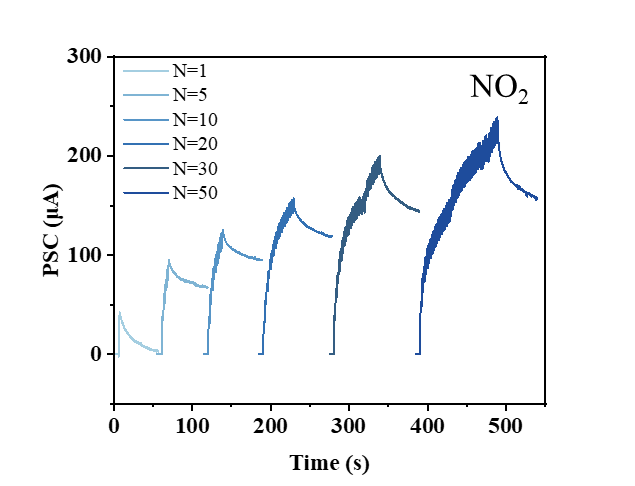


**Figure S11.** Different number of pulses (NO_2_ concentration: 100 ppm, VDS = 8 V) triggering the EPSC response.


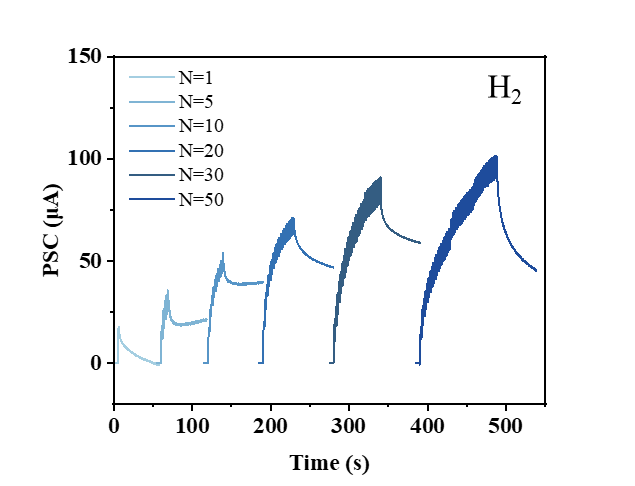


**Figure S12.** Different number of pulses (H_2_ concentration: 100 ppm, VDS = 8 V) triggering the EPSC response.


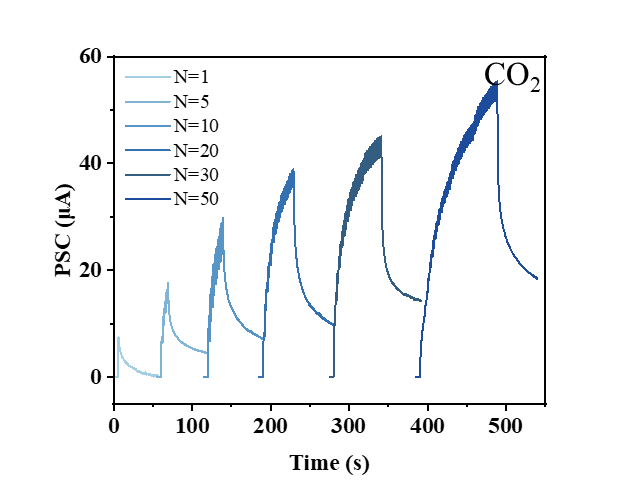


**Figure S13.** Different number of pulses (CO_2_ concentration: 100 ppm, VDS = 8 V) triggering the EPSC response.


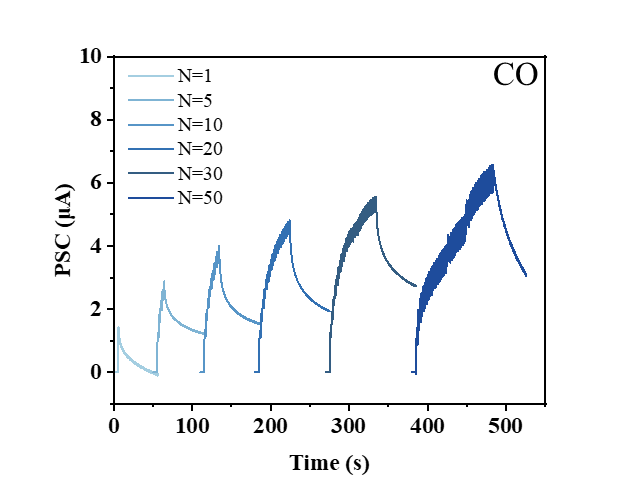


**Figure S14.** Different number of pulses (CO concentration: 100 ppm, VDS = 8 V) triggering the EPSC response.


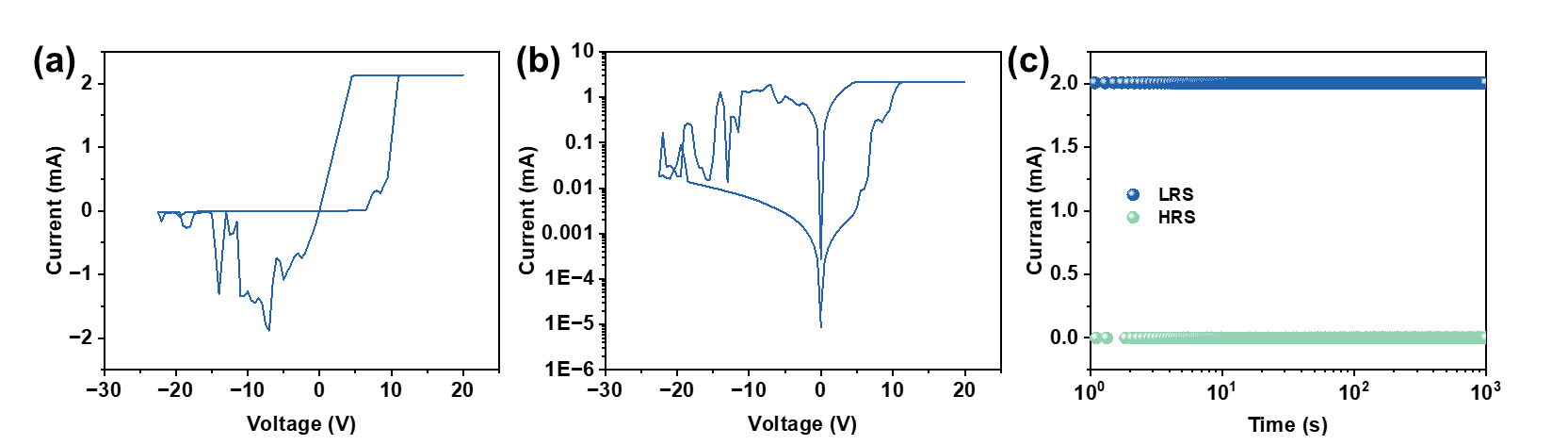


**Figure S15.** (a) I-V curve test in linear coordinates, (b) I-V curve in logarithmic coordinates, (c) maintenance characteristics of high and low configurations.


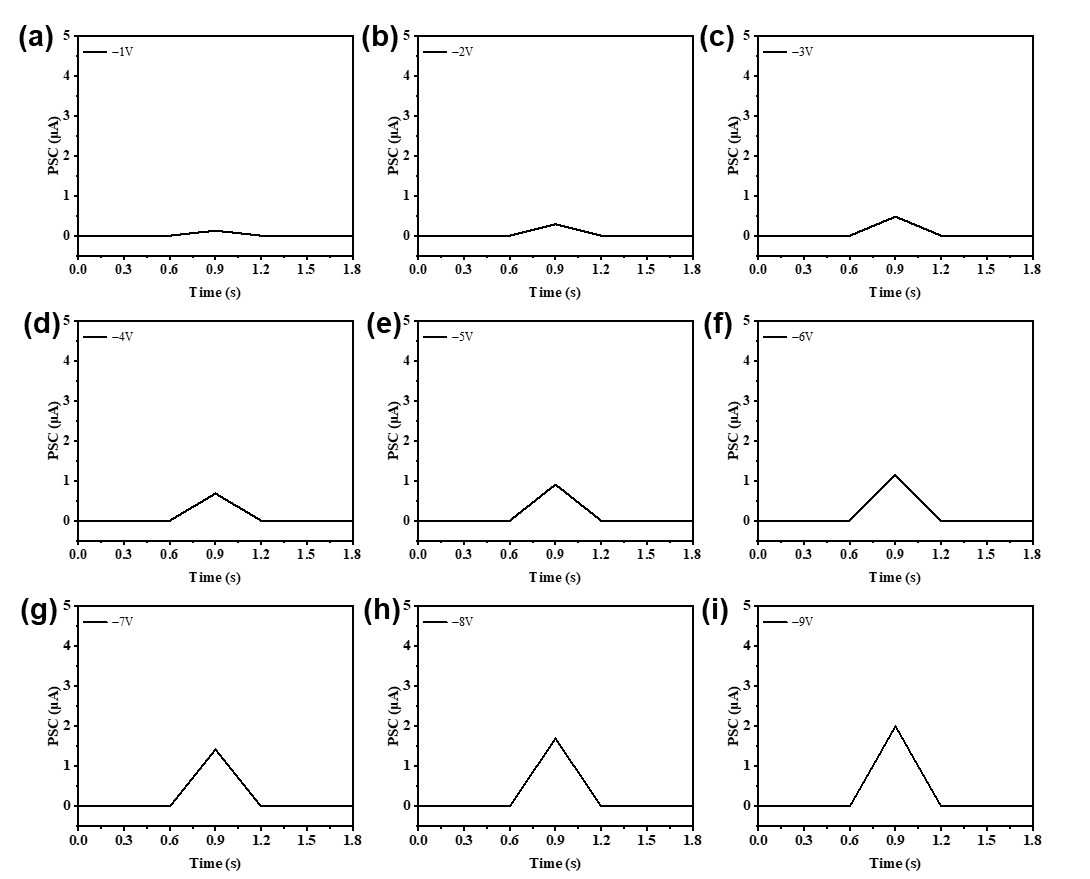


**Figure S16.** Different voltage with a pulse width of 0.3 s.


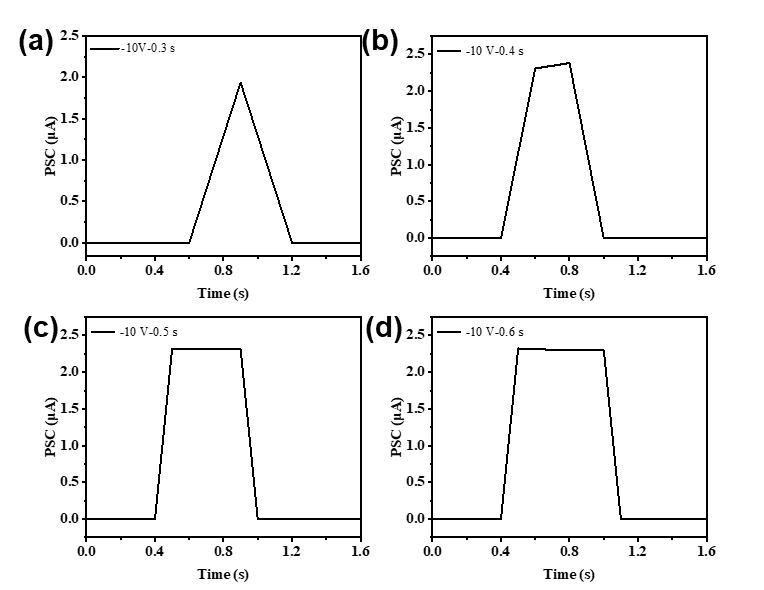


**Figure S17.** Different pulse width test at －10V.


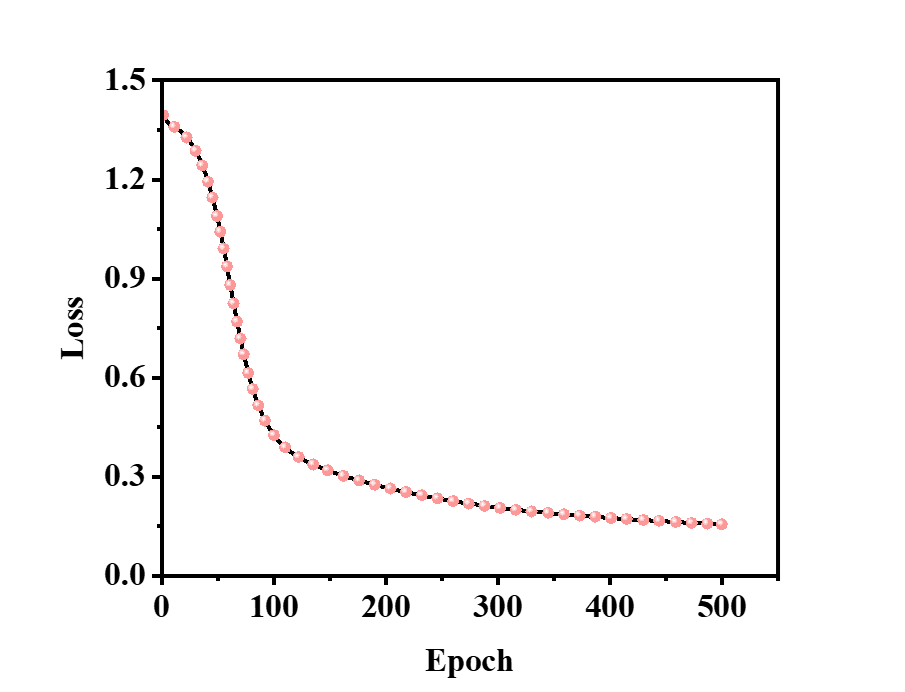


**Figure S18.** SNN loss rate curve.


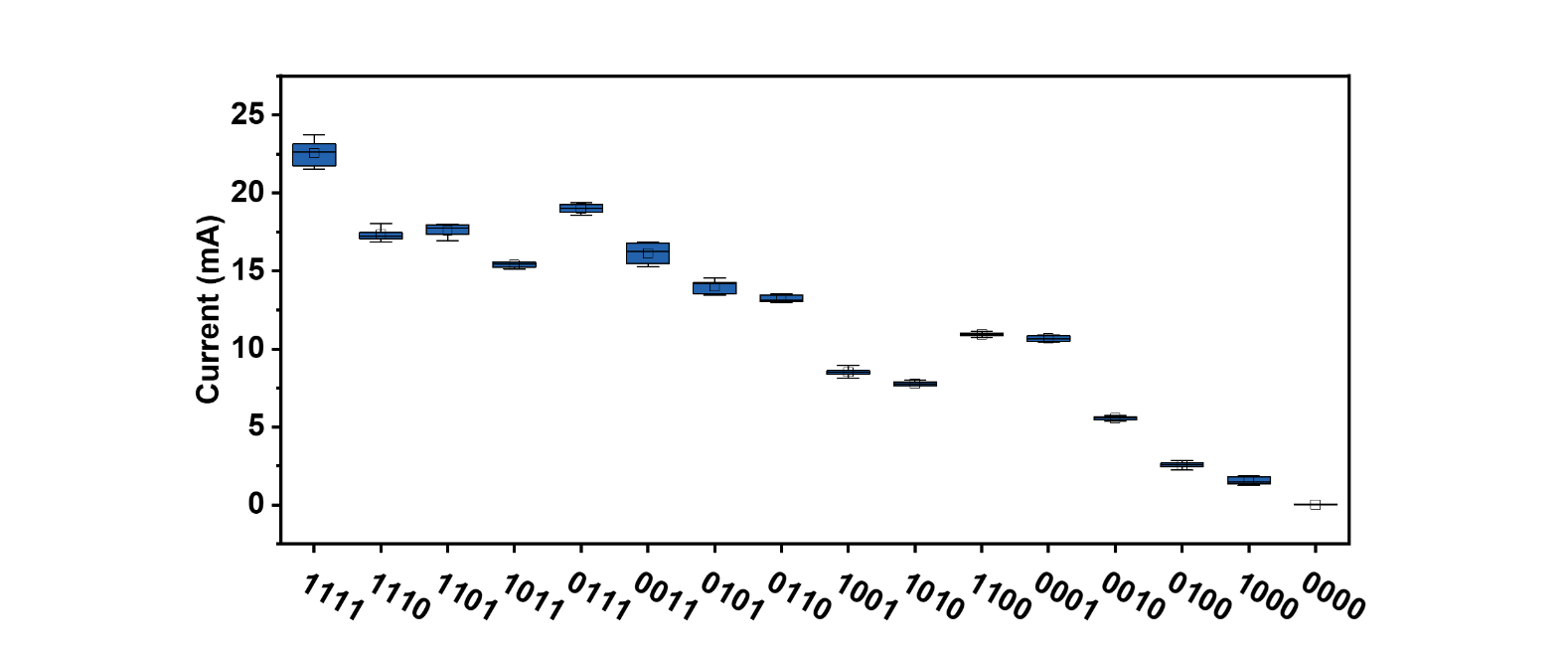


**Figure S19.** The numbers "1" and "0" represent the five-degree repeatability statistical graph.


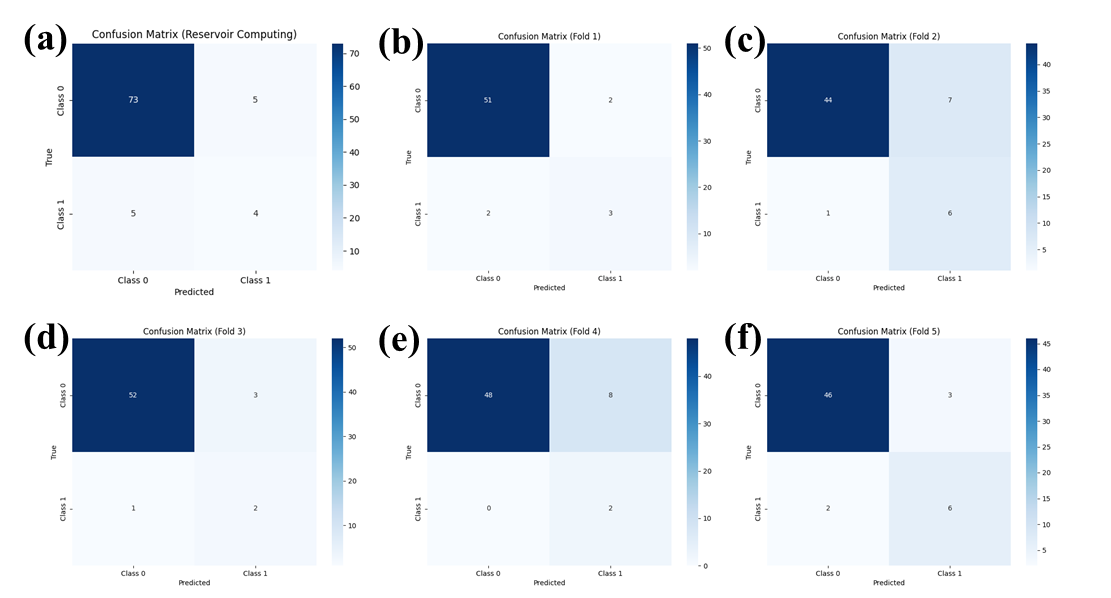


**Figure S20.** RC cross-validation training process.


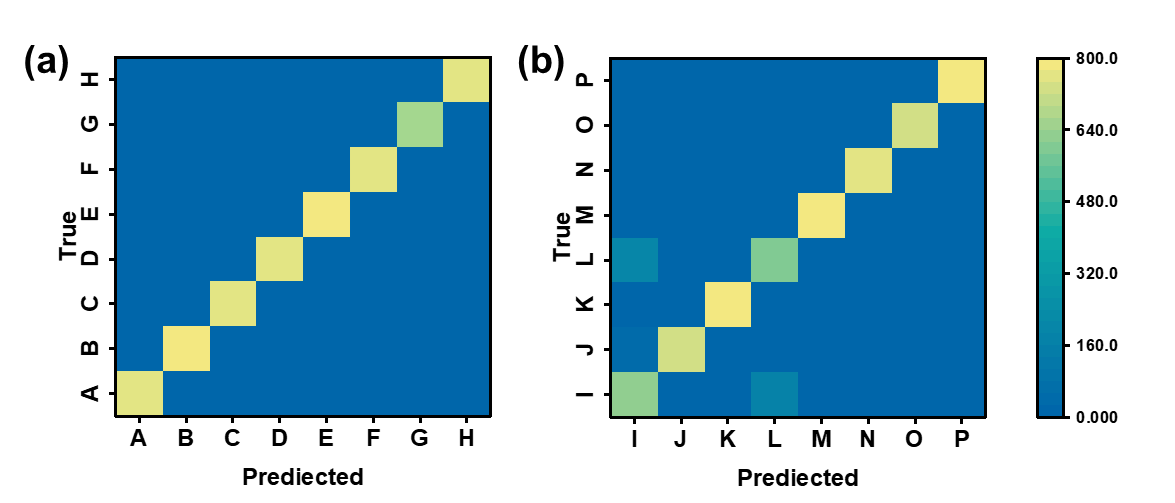


**Figure S21.** Handwriting recognition of the letters "A-P".


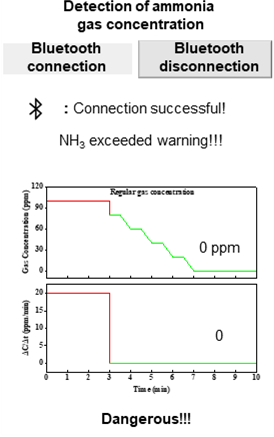


**Figure S22.** Bluetooth detection display diagram.


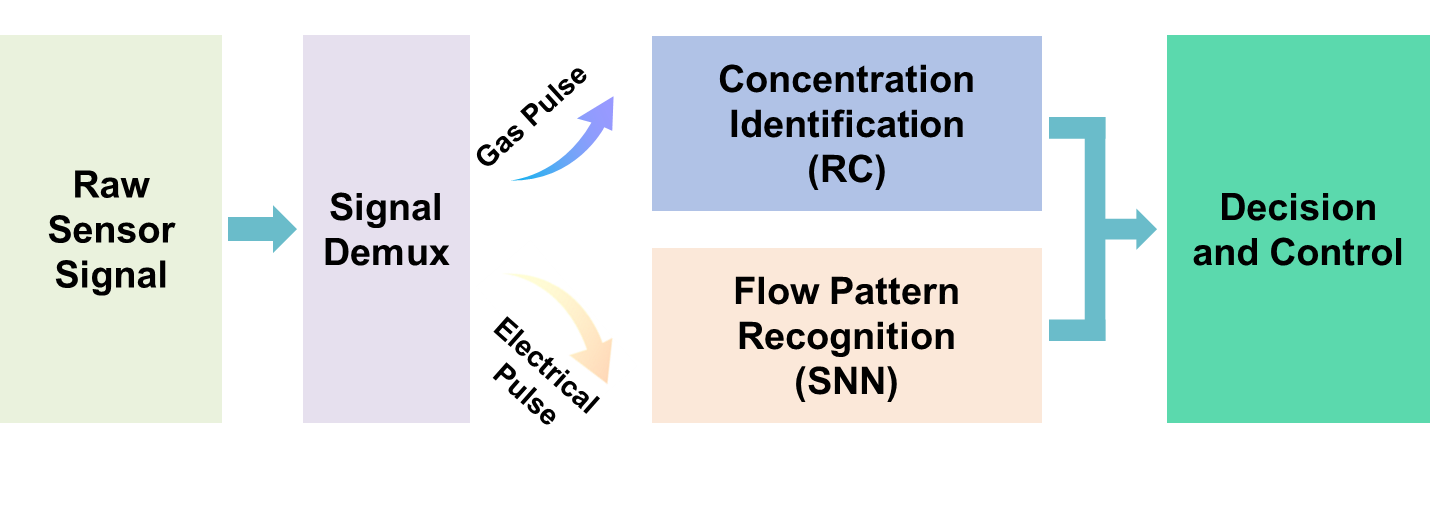


**Figure S23.** Bluetooth detection display diagram.
